# Chytrids enhance *Daphnia* fitness by selectively retained chytrid‐synthesised stearidonic acid and conversion of short‐chain to long‐chain polyunsaturated fatty acids

**DOI:** 10.1111/fwb.14010

**Published:** 2022-11-12

**Authors:** András Abonyi, Serena Rasconi, Robert Ptacnik, Matthias Pilecky, Martin J. Kainz

**Affiliations:** ^1^ WasserCluster Lunz – Biological Station Lunz am See Austria; ^2^ Centre for Ecological Research Institute of Aquatic Ecology Budapest Hungary; ^3^ Université Savoie Mont Blanc, INRAE, CARRTEL Thonon‐les‐Bains France; ^4^ Donau‐Universität Krems Krems Austria

**Keywords:** carbon transfer efficiency, fatty acid retention, mycoloop, PUFA, trophic upgrading

## Abstract

1. Chytrid fungal parasites convert dietary energy and essential dietary molecules, such as long‐chain (LC) polyunsaturated fatty acids (PUFA), from inedible algal/cyanobacteria hosts into edible zoospores. How the improved biochemical PUFA composition of chytrid‐infected diet may extend to zooplankton, linking diet quality to consumer fitness, remains unexplored.
2. Here, we assessed the trophic role of chytrids in supporting dietary energy and PUFA requirements of the crustacean zooplankton *Daphnia*, when feeding on the filamentous cyanobacterium *Planktothrix*.
3. Only *Daphnia* feeding on chytrid‐infected *Planktothrix* reproduced successfully and had significantly higher survival and growth rates compared with *Daphnia* feeding on the sole *Planktothrix* diet. While the presence of chytrids resulted in a two‐fold increase of carbon ingested by *Daphnia*, carbon assimilation increased by a factor of four, clearly indicating enhanced carbon transfer efficiency with chytrid presence.
4. Bulk carbon (*δ* ^13^C) and nitrogen (*δ* ^15^N) stable isotopes did not indicate any treatment‐specific dietary effects on *Daphnia*, nor differences in trophic position among diet sources and the consumer. Compound‐specific carbon isotopes of fatty acids (*δ* ^13^C_FA_), however, revealed that chytrids bioconverted short‐chain to LC‐PUFA, making it available for *Daphnia.* Chytrids synthesised the ω‐3 PUFA stearidonic acid *de novo*, which was selectively retained by *Daphnia*. Values of *δ* ^13^C_FA_ demonstrated that *Daphnia* also bioconverted short‐chain to LC‐PUFA.
5. We provide isotopic evidence that chytrids improved the dietary provision of LC‐PUFA for *Daphnia* and enhanced their fitness. We argue for the existence of a positive feedback loop between enhanced *Daphnia* growth and herbivory in response to chytrid‐mediated improved diet quality. Chytrids upgrade carbon from the primary producer and facilitate energy and PUFA transfer to primary consumers, potentially also benefitting upper trophic levels of pelagic food webs.

## 1 INTRODUCTION

The efficiency of dietary energy transfer across the phytoplankton–zooplankton interface is crucial for upper consumers of aquatic food webs. Traditionally, phytoplankton was considered the sole functional group supplying the dietary requirements of zooplankton (Leibold, 1989) and subsequent consumers. Zooplankton growth can be limited by multiple factors such as toxic metabolites (Ger et al., 2016), but primarily by phytoplankton biomass and biochemical dietary composition (Müller‐Navarra, 2008). The elemental composition of phytoplankton constrains zooplankton growth and reproduction in particular in case of low phosphorus concentration (Elser et al., 2001). In addition, dietary lipids and their fatty acids, especially long‐chain (LC) polyunsaturated fatty acids (PUFA) can limit zooplankton fitness (Brett et al., 2009; Müller‐Navarra et al., 2000). Reduced zooplankton fitness in response to the lack of dietary LC‐PUFA (Gulati & Demott, 1997; Ruess & Müller‐Navarra, 2019) may further constrain dietary energy and essential molecule transfer towards consumers at higher trophic levels. Accordingly, a better understanding of mechanisms that enhance PUFA transfer across the phytoplankton–zooplankton interface, when phytoplankton is limited in LC‐PUFA, is important.

High dietary quality phytoplankton, containing LC‐PUFA, include diatoms rich in eicosapentaenoic acid (EPA, 20:5 n‐3) and cryptophytes rich in EPA and docosahexaenoic acid (DHA, 22:6 n‐3; Taipale et al., 2013). The physiologically required PUFA for zooplankton are linoleic acid (LIN, 18:2 n‐6), α‐linolenic acid (ALA, 18:3 n‐3), arachidonic acid (ARA, 20:4 n‐6), EPA, and DHA. Among these dietary PUFA, EPA is particularly conductive for zooplankton growth and reproduction (Becker & Boersma, 2003; Sikora et al., 2016), while DHA increases somatic growth of fish (Copeman et al., 2002) and the cognitive abilities of consumers (Pilecky et al., 2021). Zooplankton have only a limited ability to synthesise LC‐PUFA (Arts et al., 2009; Twining et al., 2021) and lack enzymes to synthesise the essential PUFA LIN and ALA (Cook & McMaster, 2004), which therefore, have to originate from the diet. LIN and ALA are also required for potential bioconversion to ARA (i.e., LIN ➔ ARA), and to EPA via stearidonic acid (SDA, 18:4 n‐3; i.e., ALA ➔ SDA ➔ EPA), and finally to DHA (i.e., EPA ➔ DHA), respectively.

Cyanobacteria, such as *Planktothrix agardhii*, contain no LC‐PUFA (Gerphagnon et al., 2019). Accordingly, cyanobacteria blooms can limit zooplankton growth and reproduction (Gulati & Demott, 1997; Martin‐Creuzburg et al., 2009), creating an energetic bottleneck in the grazing food chain (Havens, 2008). Diet palatability can also constrain PUFA accessibility for zooplankton. Large‐sized algae or cyanobacteria, irrespective of their dietary PUFA provision, are largely inaccessible for selective (Arndt, 1993) and non‐selective filter‐feeders (Bern, 1994; Carpenter et al., 1993). Consequently, the dominance of inedible cyanobacteria (i.e., filamentous and colonial forms) can limit zooplankton growth due to both size constraints and poor diet quality, and alternative trophic pathways become crucial.

Chytrid fungal parasites constitute a key intermediate‐level trophic group between phytoplankton and zooplankton (Rasconi et al., 2014). The free‐swimming chytrid zoospores are edible (<5 μm) and constitute an alternative trophic link between large inedible phytoplankton and zooplankton, known as *mycoloop* (Kagami et al., 2007, 2014). For example, during the presence of *Asterionella*, an inedible diatom rich in LC‐PUFA, chytrids enhanced the somatic growth of *Daphnia* (Kagami et al., 2007). Similarly, chytrids infecting *Planktothrix*, an inedible cyanobacterium with poor nutritional quality, improved the growth of *Keratella* (Frenken et al., 2018) and *Daphnia* (Agha et al., 2016). Chytrids can improve the dietary provision for zooplankton in two non‐exclusive ways. First, they fragment inedible the host into smaller, edible particles (Frenken et al., 2020; Gerphagnon et al., 2013). Second, they can enhance the biochemical dietary quality compared with the uninfected hosts (Gerphagnon et al., 2019; Taube et al., 2019). Specifically, chytrids perform trophic upgrading by converting short‐chain PUFA from inedible hosts to LC‐PUFA in the edible zoospores (Gerphagnon et al., 2019; Rasconi et al., 2020; Taube et al., 2019). Chytrids also upgrade the biochemical composition of their hosts by de novo synthesised sterols, enhancing dietary energy fluxes in pelagic food webs (Gerphagnon et al., 2019). Experiments suggest that: (1) the mycoloop constitutes a quantitatively important energy pathway at the phytoplankton–zooplankton interface (Gerphagnon et al., 2019; Rasconi et al., 2020; Taube et al., 2019); and (2) chytrids enhance zooplankton consumers' fitness (Agha et al., 2016; Frenken et al., 2018). Whether and how chytrid‐mediated carbon upgrading extends to zooplankton, providing a mechanistic link between improved diet quality and consumer performance, remains unexplored.

Here we aim to disentangle the chytrid‐mediated diet quality effect, quantified by PUFA, from the increased diet quantity effect on zooplankton fitness. Using *Daphnia magna* as a herbivorous model organism, we performed a feeding experiment to compare the diets consisting of either the filamentous cyanobacterium *Planktothrix rubescens*, or *Planktothrix* infected with the obligate chytrid parasite *Rhizophydium megarrhizum* (Sønstebø & Rohrlack, 2011). We tested the hypothesis that PUFA‐induced diet quality enhancement via chytrids would improve *Daphnia* fitness, independent of the positive effect of improved diet palatability. We expected that the diet including chytrid parasites would enhance the consumer survival, growth and reproduction via zoospore consumption. Our hypothesis is based on the fact that PUFA assimilated from chytrid zoospores are among the most critical dietary biomolecules transferred across the phytoplankton–zooplankton interface, whereas their lack impedes carbon assimilation of the consumer (Taipale et al., 2014). The amount of assimilated rather than ingested carbon is the key driver of *Daphnia* fitness, which depends largely on its molecular composition, e.g. LC‐PUFA availability. The amount of assimilated carbon is the most critical in case of limiting dietary carbon availability (Khattak et al., 2018), a case highly relevant for inedible poor diet quality phytoplankton, such as the filamentous cyanobacterium *Planktothrix* (Gerphagnon et al., 2019).

To quantify the effect of improved dietary PUFA composition via the chytrid parasite, we investigated diet ingestion and assimilation of carbon. To identify PUFA bioconversion pathways among *Planktothrix*, the chytrid parasite, and *Daphnia*, we used compound‐specific stable carbon (*δ* ^13^C) isotope analysis (see Twining et al., 2020). We expected *Daphnia* to retain specific PUFA from the chytrid diet, and thus showing improved fitness compared to *Daphnia* feeding on the sole cyanobacterium.

## 2 MATERIAL & METHODS

### 2.1 Experimental cultures

The filamentous cyanobacterium *P. rubescens* (strain NIVA‐CYA97/1) and its chytrid fungal parasite (strain Chy‐Lys2009) were both isolated from Lake Lyseren, Norway (Sønstebø & Rohrlack, 2011). The cultures were maintained on WC Medium (Guillard & Lorenzen, 1972) in VWR cell culture flasks in a non‐axenic environment at 21°C. We applied 10.9 μmol m^2^ s^−1^ photosynthetically active radiation and a 16:8 light: dark cycle using two AquaLytic incubators (180 L, Liebherr, Germany). Prior to the experiment, the carbon content of uninfected and chytrid‐infected (>50% of prevalence, i.e. % of infected filaments) *Planktothrix* cultures were analysed to approximate a minimum of dietary *c*. 0.6 mg C/L during the experiment (Lampert, 1978). The high chytrid prevalence of *Planktothrix* was reached by weekly 1/3 V/V% medium exchange and 1/3 V/V% dense *Planktothrix* culture exchange. The stock cultures of *D. magna* grew in pre‐filtered (0.7 μm GF/F) water from Lake Lunz diluted with 10 V/V% of ADaM medium (Klüttgen et al., 1994) for multiple generations before the experiment. *Daphnia magna* was fed >1 mg C/L with *Scenedesmus* sp. and *Chlamydomonas* sp. (50%–50%), both grown on WC medium.

### 2.2 Experimental design

Two diet systems were compared: (1) *Planktothrix* (*cyanobacterium* treatment); and (2) chytrid‐infected *Planktothrix* (kept >50% prevalence of infection), containing the cyanobacterium, the sessile sporangia of the chytrid parasite, and free swimming chytrid zoospores (*cyanobacterium–parasite–zoospore* treatment). Diet treatments were run in five replicates in 1‐L Corning® Square Storage plastic bottles at 18°C. Each bottle contained 22 *Daphnia* neonates, all born within 48 hr. The bottles were filled up to 1 L volume (600 ml pre‐filtered LUS water + 400 ml diet containing *c*. 0.4 ± 0.1 μg C/L) and loosely covered with opaque plastic plates allowing air exchange. We applied a 16:8 light: dark cycle with 1.4 μmol m^2^ s^−1^ photosynthetically active radiation during the light phase. Food was replaced and the residuals quantified every other day to all treatments. *Daphnia* were checked daily for survival and egg production. An individual was considered dead if it was lying at the bottom of the bottle with no movement for 5 min. Dead individuals were removed and kept frozen at −20°C. The experiment was stopped when the first *Daphnia* neonates were observed (i.e., after 14 days).

### 2.3 Life history of *Daphnia*

Treatment‐specific difference in *Daphnia* growth rate was quantified based on dry weight (DW):

$$\mathrm{GR}=\left(\ln\left({\mathrm{DW}}_{\mathrm{end}}\right)-\ln\left({\mathrm{DW}}_{\text{start}}\right)\right)/d,$$

where *DW*_*end*_ is the average per individual DW at the end of the experiment, *DW*_*start*_ is the average individual‐level DW of *Daphnia* neonates, and *d* is the duration of the experiment in days. The survival of *Daphnia* was expressed as the percentage of individuals that survived the entire experiment. The egg production rate of *Daphnia* was quantified based on the cumulative number of eggs produced during the entire experiment in each bottle standardised by the number of survivals and time (per individual per day).

*Daphnia* body length was directly measured at the end of the experiment on all survival individuals according to Bottrell et al. (1976) using the ocular micrometer of a Nikon SMZ 745 T binocular microscope.

### 2.4 Quantitative data on ingested diet

The fresh‐weight (FW) biomass of ingested diet was calculated as the difference between the diet concentration at time of feeding (hereafter *diet*) and after 48 hr (hereafter *residual*) in triplicates. *Planktothrix* growth was assumed to be negligible due to the low light and water temperature compared with the culture conditions, and, therefore, not considered. Volumetric concentration of each diet source (i.e., *Planktothrix*, the chytrid‐infected *Planktothrix*, and chytrid zoospores) was quantified by microscopic analysis from formaldehyde‐fixed samples (4% final concentration). Cell density estimation from settled 3‐ml samples using a Leica DMI 3000B inverted microscope according to Utermöhl (1958) and Lund et al. (1958), with counting in at least 2 transects. Cell biovolume were approximated based on simple geometrical forms: chytrid zoospores were considered spherical (diameter of 4 μm, corresponding to 33.5 μm^3^/ind), while *Planktothrix* cells cylindrical (diameter of 3 μm, cell length of 5 μm, corresponding to 58.9 μm^3^/cell). The biovolume of *Planktothrix* filaments was calculated by multiplying the cell biovolume with the average number of cells per filaments per ml, estimated based on 20 random filaments in each sample. Ingested diet was expressed as total FW biomass assuming a density of 1. Filament lengths of *Planktothrix* was compared between the diet treatments, and also between the diets and residuals at each feeding occasion (after 48 hr) based on 30 (in case of very low densities, at least 20) random filaments. We did not include sporangia maturation in the calculation of diet ingestion for two reasons: (1) due to the high prevalence rate sessile chytrid sporangia were mainly in the size of zoospores; (2) it was technically impossible to separate the sporangia and the chytrid‐infected cyanobacterium filaments to measure their biochemical profiles separately.

Ingested *Planktothrix* and chytrid zoospores were converted into carbon based on gravimetrically measured DW (KERN, ABT 220‐5DM) of the seston provided, the C% of diet sources and the respective % of FW biomass ingested. We also calculated ingestion efficiency for each diet source based on the ratio of the ingested and provided FW biomass (i.e. the dietary biomass % ingested).

Bacteria were also quantified in the diet sources and their residuals to estimate bacterial carbon ingestion using a Cytoflex AS18172 (Backman Coulter) flow cytometer. The formaldehyde‐fixed (4% final concentration) samples were filtered with a 2‐ μm mesh (CellTrics™) to remove *Planktothrix* filaments, sonicated for 20 min, 100× diluted and then stained with the fluorescent acidotropic probe LysoSensor™ Green (Invitrogen™, Fisher Scientific). The samples were run at 60 μl/min flow rate for at least 180 s with <500 events/s and analysed with the CytExpert (v.2.1) software by manual gating to detect bacterial cells with positive LysoSensor™ signal. Bacterial abundance was converted to bacteria carbon by assuming 20 fg C/cell (Agha et al., 2016; Ducklow & Carlson, 1992).

### 2.5 Elemental C, N, P, and stable C and N isotope analyses

The elemental C and N contents as well as the stable isotopes of carbon (*δ* ^13^C) and nitrogen (*δ* ^15^N) of diet sources were analysed during three occasions in triplicates (*n* = 9): before the experiment, immediately after the experiment, and 1 week after the end of the experiment. Previous data on the same cultures showed that the sole *Planktothrix* varied less in C%, N%, and lipid content compared with the chytrid zoospores (Rasconi et al., 2020). In a preliminary experiment, a large variation in C% and N% of the chytrid‐infected *Planktothrix and* chytrid zoospores (Appendix S1) was also observed. Here the sole *Planktothrix* diet was analysed only once in triplicates (*n* = 3) right before the experiment. *Daphnia* were analysed for C, N, and P in triplicates, both at the beginning (*Daphnia* neonates; time point zero, T_0_) and at the end of the experiment (time point end, T_E_).

To separate the chytrid zoospores from *Planktothrix* filaments, *c*. 100 ml culture was filtered five times through a 20‐μm mesh followed by a single filtration step through a 5‐μm mesh. Absence of *Planktothrix* in chytrid zoospore samples was evaluated using a Nikon Eclipse TS100 inverted microscope at 200× magnification.

Individual diet sources were collected on muffled and pre‐weighed GF/F Whatman™ filters (25 mm, 0.7 μm pore size), dried for 48 hr at 50°C (oven MMM Ecocell), weighed (KERN, ABT 220‐5DM) and folded in tin capsules (IVA, Analys. GmbH & Co. KG) with *c*. 0.3 mg DW seston. *Daphnia* were frozen at −80°C and subsequently freeze‐dried for 48 hr (VirTis benchtopK, VWR), weighed, and folded in tin capsules *c*. 0.3 mg DW. C, N, and bulk carbon (*δ* ^13^C) and nitrogen (*δ* ^15^N) stable isotopes were measured using a Thermo Fisher Scientific Flash 2000 Elemental Analyser, linked to a Delta V Advantage Mass Spectrometer. *Δ* ^13^C values were referenced to the Vienna PeeDee Belemnite (^13^C:^12^C = 0.01118), using USGS standards, applying the formula:

$$\delta^{13}C_{\mathrm{FA}}=\left(\frac{{}^{13}C{/}^{12}C_{\text{Sample}}}{{}^{13}C{/}^{12}C_{\text{VPDB}}}-1\right)\times 1000$$

To rule out a potential dietary effect of P over PUFA in *Daphnia*, we quantified POP in *Daphnia* at T_0_ and T_E_ based on the ascorbic acid colorimetric method following persulfate digestion (American Public Health Association, 1998). P was not measured in diet sources during the experiment, but P was never limiting in culture conditions (i.e. C:P < 50 in all diet sources, independent measure of the experiment, data not shown). Elemental ratios expressed as molar ratios were C:N for diet sources, and C:N, C:P and N:P for *Daphnia*.

### 2.6 Fatty acid and compound‐specific carbon isotope analyses

The fatty acid composition and their compound‐specific carbon isotopes (*δ* ^13^C_FA_) were analysed in the diet treatments in line with the elemental composition. Individual diet sources (*c*. 400 ml of culture, *c*. 1 mg seston; DW) were collected on muffled and pre‐weighed GF/F Whatman™ filters (47 mm, 0.7 μm pore size). *Daphnia* were analysed for FA and *δ* ^13^C_FA_ profiles at the beginning (T_0_) and at the end (T_E_) of the experiment in triplicates. *Daphnia* were freeze‐dried (see above) and *c*. 1 mg DW was transferred into tin caps.

Lipids were extracted from the freeze‐dried, homogenised samples using chloroform–methanol mix (2:1), following the protocol described in Heissenberger et al. (2010). FA were derivatised to methyl esters by incubation with 1% H_2_SO_4_ in methanol at 50°C for 16 hr. FA methyl esters (FAMEs) were dried under N_2_ and dissolved in hexane. For quantification via flame ionisation, FAMEs were separated using a GC (Trace™ 1,310 Thermo Specific, Italy) equipped with a Supelco™ SP‐2560 column (100 m × 0.25 mm × 0.2 μm). ^13^C isotope analysis was performed using a Thermo Trace 1,310 GC (ThermoFisher Scientific), connected via a ConFlo IV (Thermo Co.) to an isotope ratio mass spectrometer (DELTA V Advantage, Thermo Co.). FAMEs were separated using a VF‐WAXms 60‐m column, 0.25 mm ID, film thickness 0.25 μm following oxidation to CO_2_ in a combustion reactor, filled with Ni, Pt, and Cu wires, at a temperature of 1,000°C. The temperature started at 80°C, which was kept for 2 min, after which the temperature was raised by 30°C/min to 175°C, by 5°C/min to 200°C and finally by 2.4°C/min to 250°C, which was maintained for 30 min. Samples were run against certified Me‐C20:0 standards (USGS70: δ^13^C = −30.53‰, USGS71: δ^13^C = −10.5‰, and USGS72: δ^13^C = −1.54‰), which were used for drift and linear correction. All peaks were validated and corrected manually if necessary.

### 2.7 Carbon transfer efficiency

Carbon transfer efficiency was calculated as the ratio of carbon accrual by *Daphnia* (i.e., the total weight gained in carbon) to the total ingested carbon (Müller‐Navarra et al., 2000), and expressed as %: 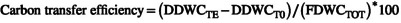, where *DDWC* is the average DW of *Daphnia* expressed in carbon in each replicate at the end (T_E_) and start of the experiment (T_0_), *FDWC*_*TOT*_ is the total DW of ingested diet in carbon. Total ingested carbon was calculated from ingestion rates, standardised by the number of surviving *Daphnia* individuals at each feeding occasion (i.e. accounting for the change in abundances per replicate). *Compound‐specific* ^*13*^ *C PUFA pathways*. We quantified each *δ* ^13^C PUFA value to follow dietary PUFA sources for *Daphnia* by calculating the difference in *δ* ^13^C of each FA between the consumer (*δ* ^13^C_C_) and its respective diet sources (*δ* ^13^C_D_), i.e. Δ^13^C_FA_, following Chiapella et al. (2021): Δ^13^C_FA_ = δ^13^C_C_ − δ^13^C_D_. In a similar way, we calculated Δ^13^C_FA_ differences within *Daphnia* between LIN ➔ ARA, ALA ➔ SDA, ALA ➔ EPA, and SDA ➔ EPA conversion pathways.

### 2.8 Data analyses

Treatment‐specific differences in *Daphnia* growth rates, egg production rates, survival, and carbon transfer efficiency were tested with one‐way ANOVA, or Wilcoxon in the case of unequal variance. Ingested diet among sources, as well as the C:N, C:P and N:P ratios in *Daphnia* were compared with two‐way ANOVA with multiple comparisons of means (function *glht* using Tukey's contrasts in the *multcomp* R package). The statistically significant value was set at *p* < 0.05.

To visualise ingestion efficiency of diet sources by *Daphnia* over time, linear [LM] and non‐linear (generalised additive models [GAMs] with smooth splines, Wood, 2011, 2017) regressions were fitted to the time trends. The best model was selected according to the Akaike information criterion. Pearson's product–moment correlation analysis was also performed to test how the number of eggs produced by *Daphnia* was related to body length (size) at the end of the experiment.

Individual FA, total FAME, and PUFA among diet sources and *Daphnia* were analysed by ANOVA, reporting Tukey's HSD. The statistically significant value was set at *p* < 0.05. Fatty acid data were based on mass fractions (μg FA/mg DW) and log (x + 1) transformed to improve normality.

A redundancy analysis ordination was applied to relate the FA profiles of *Daphnia* to the FA profile of diet sources in a quantitative way, i.e. using diet FA profiles as independent constraining variables. To this end, we weighted the FA matrix of *Daphnia* by their DW gained (DW_TE_ – DW_T0_), and the FA matrix of diet sources by their respective total amount of DW ingested. The FA matrices were ln (x + 1) transformed, and significant dietary FA selected in an a priori way based on combined backward and forward selection (function *ordistep* in *vegan*; Oksanen et al., 2019). The final significance of models was tested by 999 Monte Carlo permutations for each term in full models (function *ANOVA*; by = *terms*). All data analyses and visualisations were performed in R (R Core Team, 2019).

## 3 RESULTS

### 3.1 Life history of *Daphnia*

**FIGURE 1:**
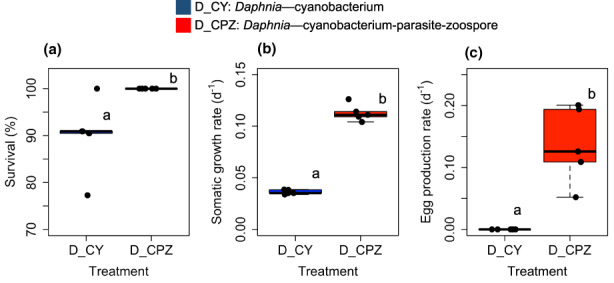
(a) Survival of *Daphnia* (in %) during the experiment; (b) somatic growth rates of *Daphnia* based on dry weight; (c) egg production rates of *Daphnia*. D_CY: *Daphnia* feeding on the cyanobacterium (*Planktothrix*), D_CPZ: *Daphnia* feeding on the chytrid‐infected cyanobacterium (*Planktothrix*, chytrid parasites and chytrid zoospores). Letters indicate the level of significance between diet treatments based on ANOVA (*n* = 5, at *p* < 0.05).

The survival of *Daphnia* was significantly higher in the chytrid‐infected diet treatment (Figure 1a, Wilcoxon, W = 2.5, *n* = 5, *p* < 0.05). The somatic growth rate of *Daphnia* feeding on chytrid‐infected *Planktothrix* was also significantly higher compared with *Daphnia* feeding on the sole *Planktothrix* diet (Figure 1b, ANOVA, *F*_1,4.5_ = 404.6, *n* = 5, *p* < 0.001). *Daphnia* produced eggs only in the chytrid‐infected *Planktothrix* diet treatment (Figure 1c, Wilcoxon, W = 0, *n* = 5, *p* < 0.01). The number of eggs produced by *Daphnia* in the chytrid‐infected *Planktothrix* diet treatment correlated moderately, but significantly with body lengths (Pearson, *r*[104] = 0.43, *p* < 0.001).

### 3.2 Filament length of *Planktothrix*

The length of *Planktothrix* did not differ significantly between uninfected (range: 30–1,000 μm, median: 530 μm) and infected (range: 30–6,000 μm, median: 460 μm) filaments between the supplied diet treatments (Wilcoxon, W = 11,158, *p* > 0.05). The length of *Planktothrix* supplied as diet did not vary over time (GAM, $R_{\mathrm{Adj}}^{2}$ = −0.0008, *p* > 0.05, Appendix S1), while it significantly decreased with time in its residual (GAM, $R_{\mathrm{Adj}}^{2}$ = 0.12, *p* < 0.001). The length of chytrid‐infected *Planktothrix* supplied as diet decreased slightly over time (GAM, $R_{\mathrm{Adj}}^{2}$ = 0.05, *p* < 0.05; Appendix S1), while in the residual, it decreased considerably (GAM, $R_{\mathrm{Adj}}^{2}$ = 0.26, *p* < 0.001). The residual of the chytrid‐infected *Planktothrix* diet (range: 10–1,300 μm, median: 400 μm) was significantly shorter compared with the residual of the uninfected *Planktothrix* diet (range: 15–10,000 μm, median: 200 μm; Wilcoxon, W = 180,637, *p* < 0.001).

The prevalence of chytrids in the chytrid‐infected *Planktothrix* diet stayed >50% during the experiment, and it decreased linearly with time in the residual of diet collected after 48 hr (Appendix S1). The difference in the prevalence of chytrids between the diet provided and its residual increased over time, i.e. a decreasing negative trend in ∆ (LM, $R_{\mathrm{Adj}}^{2}$ = 0.715; *p* < 0.001, Appendix S1).

### 3.3 Ingestion rates

**FIGURE 2:**
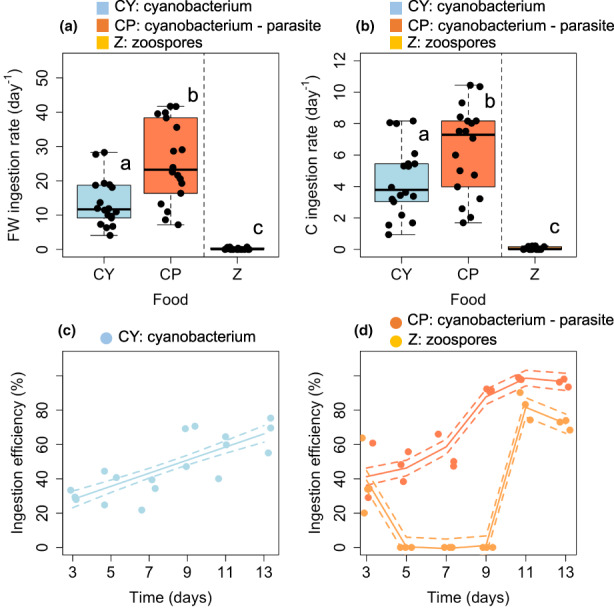
(a) Ingestion rates of diet sources expressed as fresh weight (FW): CY—Cyanobacterium (*Planktothrix*), CP—Chytrid‐infected cyanobacterium, Z—Chytrid zoospores; (b) ingestion rates of diet expressed as carbon; (c) ingestion efficiency (% of diet biomass ingested) by *Daphnia* for *Planktothrix* with fitted linear trend line (LM); (d) ingestion efficiency of the chytrid‐infected *Planktothrix* and chytrid zoospores by *Daphnia* with fitted non‐linear trend lines (generalised additive model). Letters indicate the level of significance among diet sources based on ANOVA, multiple comparison of means, Tukey (*n* = 18, *p* < 0.05).

The ingestion rate of FW diets per *Daphnia* differed significantly among diet sources (Figure 2a). *Daphnia* ingested *c*. 2× more FW biomass of the chytrid‐infected *Planktothrix* (25 ± 12 μg ind^−1^ day^−1^) than of the uninfected *Planktothrix* (14 ± 7 μg ind^−1^ day^−1^), and comparatively, much less biomass of chytrid zoospores compared with of the chytrid‐infected and non‐infected *Planktothrix* (0.2 ± 0.3 μg ind^−1^ day^−1^). *Daphnia* ingested 6.4 ± 2.8 μg C ind^−1^ day^−1^ of chytrid‐infected *Planktothrix* (median: 7.3 μg C ind^−1^ day^−1^), 4.4 ± 2.2 μg C ind^−1^ day^−1^ of *Planktothrix* only (median: 3.8 μg C ind^−1^ day^−1^), and 0.07 ± 0.004 μg C ind^−1^ day^−1^ of chytrid zoospores (median: 0.02 μg C ind^−1^ day^−1^), with significant differences among diet sources (Figure 2b, *p*_AP–A_ < 0.05, *p*_A–Z_ < 0.001, *p*_AP–Z_ < 0.001). *Daphnia* ingested 7.2*10^5^ ± 2.3*10^5^ bacteria ind^−1^ day^−1^ in the *Planktothrix* diet treatment, corresponding to 0.37 ± 0.12 μg C ind^−1^ day^−1^. *Daphnia* in the chytrid‐infected *Planktothrix* diet treatment ingested 1.5*10^6^ ± 6.7*10^5^ bacteria ind^−1^ day^−1^, corresponding to 0.69 ± 0.31 μg C ind^−1^ day^−1^.

The ingestion efficiency of *Planktothrix* increased significantly and linearly with time in the *Planktothrix*‐only treatment (Figure 2c, LM, $R_{\mathrm{Adj}}^{2}$ = 0.5622, *p* < 0.001), while it increased exponentially, levelling off towards the end of the experiment in the chytrid‐infected *Planktothrix* treatment (Figure 2d, GAM, $R_{\mathrm{Adj}}^{2}$ = 0.881, *p* < 0.001). The chytrid zoospore density supplied in chytrid‐infected diet ranged between 253 and 1,214 ind/ml (median: 678 ind/ml), which was grazed with varying efficiency. The ingestion efficiency of chytrid zoospores first dropped from *c*. 40% to no grazing (0%) for the period from day 5 to 9, then increased with time, where its maxima (*c*. 70%–80%) occurred at the end of the experiment (Figure 2d, GAM, $R_{\mathrm{Adj}}^{2}$ = 0.929, *p* < 0.001).

### 3.4 Stoichiometry of diet sources and *Daphnia*

Chytrid zoospores had significantly higher C content than the chytrid‐infected *Planktothrix*, while C in chytrid‐infected and uninfected *Planktothrix* did not differ significantly (Appendix S1, ANOVA, multiple comparisons of means, Tukey, *p*_Z–AP_ <0.01, and *p* > 0.05 in all other cases). The N content of chytrid zoospores was significantly higher compared with the uninfected (*p*_Z–A_ < 0.01) and chytrid‐infected *Planktothrix* (*p*_Z–AP_ <0.001), while it did not differ significantly between chytrid‐infected and uninfected *Planktothrix* (ANOVA, multiple comparisons of means, *p*_A–AP_ >0.05, in all other cases). The C:N ratio among diet sources differed only significantly between chytrid‐infected *Planktothrix* and chytrid zoospores (ANOVA, multiple comparisons of means, Tukey, *p*_Z–AP_ <0.05).

In *Daphnia*, the C, N, and P contents did not differ significantly among *Daphnia* at T_0_ and T_E_, nor at T_E_ between diet treatments (Appendix S1, multiple comparisons of means, Tukey, *p* > 0.05, in all cases). The C:N ratio did not differ significantly between *Daphnia* feeding on *Planktothrix* and chytrid‐infected *Planktothrix* diets (ANOVA, Tukey, *p* > 0.05), while it was significantly lower in *Daphnia* feeding on both experimental diets compared to *Daphnia* neonates (ANOVA, Tukey, *p*_N–A_ < 0.01, *p*_N–APZ_ <0.05). The C:P ratio did not differ significantly among *Daphnia* neonates or adults with any treatment (ANOVA, Tukey, *p* > 0.05, in all cases). The N:P ratio was significantly higher in *Daphnia* feeding on the *Planktothrix* diet compared to *Daphnia* neonates (ANOVA, Tukey, *p*_N–A_ < 0.05, in all cases), while all the other pairwise comparisons were non‐significant (ANOVA, Tukey, *p* > 0.05, in all cases).

### 3.5 Bulk *δ* ^13^C and *δ* ^15^ N values of diet sources and *Daphnia*

The *δ* ^13^C values differed only significantly between *Planktothrix* and chytrid zoospores, with zoospores having significantly lower values (Appendix S1, Wilcoxon, W = 0, *p* < 0.05), and between *Daphnia* neonates and chytrid zoospores, with zoospores having significantly lower values (Wilcoxon, W = 0, *p* < 0.05). The *δ* ^15^N did not differ significantly among diet sources or among diet sources and *Daphnia* consumers (Wilcoxon, *p* > 0.05 in all cases). The *SD* of both *δ* ^13^C and *δ* ^15^N values were high, especially in the chytrid‐infected diet, and in *Daphnia* feeding on the chytrid‐infected diet.

### 3.6 Fatty acid contents of diet sources and *Daphnia*

Total lipids in *Daphnia*, both at the beginning and end of the experiment, were significantly higher than in diet sources (ANOVA, *n* = 30, *F*_1,5_ = 37, *p* < 0.001). Total PUFA content was also highest in *Daphnia* neonates (107 ± 8 μg/mg, Tukey's HSD; *p* ≤ 0.01), higher than in *Daphnia* feeding on chytrid‐infected *Planktothrix* (17 ± 3 μg/mg) and *Planktothrix* alone (16 ± 0.5 μg/mg).

**FIGURE 3:**
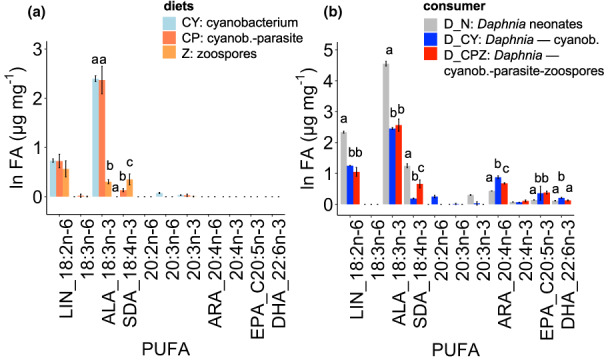
(a) Polyunsaturated fatty acids (PUFA) content of diet sources. CY: Cyanobacterium (*Planktothrix*), CP: Cyanobacterium‐chytrid parasite, Z: Chytrid zoospores; (b) PUFA content of *Daphnia* at the beginning of the experiment (D_N: *Daphnia* neonates) and at the end of the experiment for only *Planktothrix* diet (D_CY) and for chytrid‐infected *Planktothrix* diet (D_CPZ) including zoospores. Letters denote the level of significance among diet sources, *Daphnia* neonates, and *Daphnia* in the different diet treatments based on ANOVA, multiple comparison of means, Tukey (*p* < 0.05).

Dietary PUFA content was identical between *Planktothrix* and chytrid‐infected *Planktothrix* (11 ± 0.7 μg/mg and 11 ± 3 μg/mg, respectively), and significantly lower in chytrid zoospores (Tukey's HSD; *p* < 0.01). Diet sources were identical in terms of short‐chain PUFA content (Figure 3a), except chytrid zoospores having the smallest content of ALA (Tukey's HSD; *p* < 0.001). Among dietary LC‐PUFA, the SDA content was the highest in chytrid zoospores (0.4 ± 0.16 μg/mg), followed by chytrid‐infected *Planktothrix* (0.1 ± 0.03 μg/mg), while it was absent in *Planktothrix*. Diet sources did not contain LC‐PUFA, such as ARA, EPA, or DHA.

Short‐chain PUFA in *Daphnia* decreased significantly between neonates and adults at the end of the experiment, while LC‐PUFA increased (Figure 3b). *Daphnia* feeding on chytrid‐infected *Planktothrix* had significantly higher SDA (0.9 ± 0.2 μg/mg) compared with *Daphnia* feeding on uninfected *Planktothrix* (0.2 ± 0.03 μg/mg, Tukey's HSD; *p* < 0.05). *Daphnia* adults at the end of the experiment contained ARA, EPA, and DHA. ARA was significantly higher in *Daphnia* feeding on chytrid‐infected *Planktothrix* (1.4 ± 0.1 μg/mg) compared to *Daphnia* feeding on *Planktothrix* (1 ± 0.04 μg/mg). The EPA content was significantly higher in *Daphnia* adults irrespective of diet treatments compared with *Daphnia* neonates (Tukey's HSD; *p* < 0.001). The DHA content was significantly higher in *Daphnia* feeding on the sole *Planktothrix* diet (Tukey's HSD; *p* < 0.001) compared with *Daphnia* feeding on the chytrid‐infected diet.

The total bacterial fatty acid content differed significantly among diet sources (ANOVA, *F*_1,2_ = 23.91, *p* < 0.001). The chytrid‐infected *Planktothrix* diet contained >2× more bacterial fatty acids (10.2 ± 2.4 μg/mg; measured in the chytrid zoospore fraction) than the uninfected *Planktothrix* diet (3.9 ± 1.5 μg/mg). The total bacterial fatty acid content in *Daphnia* did not differ significantly between diet treatments (ANOVA, multiple comparisons of means, Tukey, *p* > 0.05), and decreased significantly in *Daphnia* feeding on both diet treatments (*Planktothrix* diet: 5.2 ± 0.2 μg/mg, chytrid‐infected *Planktothrix* diet: 7.3 ± 1.8 μg/mg) compared with *Daphnia* neonates (19.3 ± 1.3 μg/mg; ANOVA, multiple comparisons of means, Tukey, *p* < 0.001, in both cases).

### 3.7 Carbon transfer efficiency related to PUFA

**FIGURE 4:**
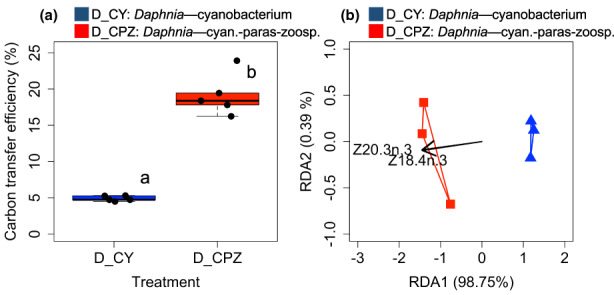
(a) Carbon transfer efficiency to *Daphnia* based on the ratio of carbon accrual and ingested carbon from diet sources (D_CY: *Daphnia* feeding on the cyanobacterium (*Planktothrix*), D_CPZ: *Daphnia* feeding on chytrid‐infected *Planktothrix*, *n* = 5). Letters indicate the level of significance between *Daphnia* in the different diet treatments (ANOVA; *p* < 0.05); (b) redundancy analysis (RDA) ordination predicting the FA profile of *Daphnia* (weighted by carbon accrual) from the FA profiles of diet sources (weighted by their respective carbon weight digested). Full model: *p* < 0.05; axes: *p*_RDA1_ < 0.05, *p*_RDA2_: n.s.

The carbon transfer efficiency was *c*. 4× higher in *Daphnia* feeding on chytrid‐infected *Planktothrix* (19.2 ± 2.9%) compared with *Daphnia* feeding on the sole *Planktothrix* diet (4.9 ± 0.4%, Figure 4a, ANOVA, *F*_1,4.1_ = 119, *n* = 5, *p* < 0.001).

The fatty acid composition of diet sources, weighted by their respective DW ingested, explained 99.1% variance in the FA profile of *Daphnia*, weighted by their DW gained (Figure 4b, redundancy analysis, *p*[ANOVA] < 0.05). Two dietary FA affected the FA profile of *Daphnia* significantly: SDA and 20:3 n‐3, both originating from chytrid zoospores (*p*[adonis] < 0.01).

### 3.8 Compound specific carbon isotopes of PUFA in *Daphnia*

**FIGURE 5:**
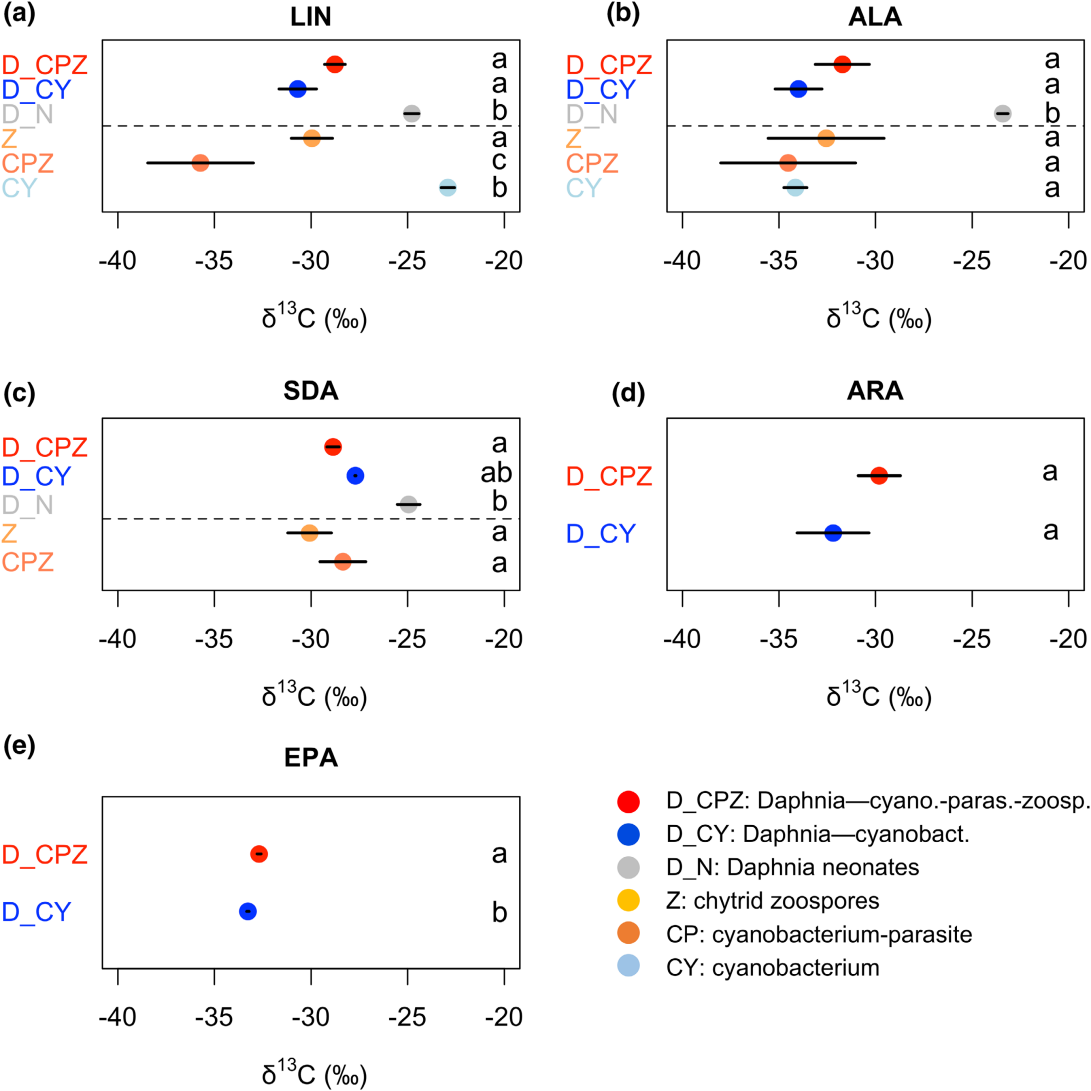
Mean ± *SD* of the *δ* ^13^C signal in polyunsaturated fatty acids (PUFA) in the CY—Cyanobacterium (*Planktothrix*), in the chytrid‐infected *Planktothrix* (CP—Cyanobacterium‐chytrid parasite), chytrid zoospores (Z), and in *Daphnia* neonates (D_N), *Daphnia* feeding on cyanobacterium (D_CY) and the on the chytrid‐infected cyanobacterium (D_CPZ); (a) the short‐chain ω‐6 PUFA linoleic acid (LIN); (b) the short‐chain ω‐3 PUFA alpha‐linoleic acid (ALA); (c) the long‐chain ω‐3 PUFA stearidonic acid (SDA); (d) the long‐chain ω‐6 PUFA arachidonic acid (ARA); (e) the long‐chain ω‐3 PUFA eicosapentaenoic acid (EPA). Letters indicate the level of significance among diet sources, *Daphnia* neonates, and *Daphnia* in the different diet treatments based on ANOVA, multiple comparison of means, Tukey (*p* < 0.05). Dotted vertical lines separate the fatty acid profiles of diets and *Daphnia*.

The *δ* ^13^C_LIN_ values differed significantly among diets and *Daphnia* (Figure 5a). Among the diets, LIN was isotopically the most enriched in *Planktothrix*, significantly lighter in the chytrid‐infected *Planktothrix* (*p* < 0.001), and significantly heavier again in chytrid zoospores (*p* < 0.05, ANOVA, multiple comparisons of means, Tukey). The *δ* ^13^C_LIN_ values did not differ significantly in *Daphnia* between the two diet treatments, while they were significantly heavier in *Daphnia* feeding on the chytrid‐infected diet compared with the diet itself (*p* < 0.01, ANOVA), but not compared with chytrid zoospores (*p* > 0.05, ANOVA). The *δ* ^13^C_ALA_ values did not differ significantly either among diet sources, or among diet sources and *Daphnia* from the diet treatments, or in *Daphnia* between the two diet treatments (*p* > 0.05 in all cases, ANOVA, Figure 5b). The δ^13^C_SDA_ values were significantly lighter in chytrid zoospores (*p* < 0.01, ANOVA), in the chytrid‐infected *Planktothrix* (*p* < 0.05, ANOVA), and in *Daphnia* feeding on the chytrid‐infected *Planktothrix* (*p* < 0.01, ANOVA) compared with *Daphnia* neonates (Figure 5c). The *δ* ^13^C_ARA_ values did not differ significantly in *Daphnia* among diet treatments, but *δ* ^13^C_EPA_ did differ significantly with higher values in *Daphnia* feeding on the chytrid‐infected diet (ANOVA, multiple comparisons of means, Tukey, *p* < 0.01, Figure 5d,e).

The Δ^13^C discrimination factor of individual PUFA indicated that *Daphnia* feeding on *Planktothrix* were significantly depleted in δ^13^C_LIN_ (−7.8‰), while slightly enriched in δ^13^C_ALA_ (+0.2‰). *Daphnia* feeding on the chytrid‐infected *Planktothrix* were enriched in both δ^13^C_LIN_ (+6.9‰) and δ^13^C_ALA_ (+2.8‰)_,_ although the difference was only significant for LIN. Comparing *Daphnia* feeding on chytrid zoospores, the consumer was slightly enriched in all δ^13^C_LIN_ (+1.2‰), δ^13^C_ALA_ (+0.8‰) and δ^13^C_SDA_ (+1.2‰) from the zoospores, but the differences were not significant.

Since SDA was missing in the *Planktothrix* diet, as well as ARA and EPA in *Daphnia* neonates, we calculated the Δ^13^C discrimination factor to compare *δ* ^13^C of SDA, ARA, AND EPA with *δ* ^13^C of their precursors: LIN, ALA, and SDA (Table 1). Δ^13^C_ARA_ indicated slightly depleted *δ* ^13^C in the consumer compared with its precursor LIN, while the difference was not significant for either diet treatments. Consumers’ SDA was enriched with *δ* ^13^C compared to *δ* ^13^C_ALA_ in both diet treatments, while it was only significant in *Daphnia* feeding on *Planktothrix*. *Daphnia* feeding on the *Planktothrix* diet was largely depleted in *δ* ^13^C_EPA_ compared to *δ* ^13^C_SDA_, while compared to both *δ* ^13^C_ALA_ and *δ* ^13^C_SDA_ in the chytrid‐infected diet treatment.

**TABLE 1:** Discrimination factors (Δ^13^C) between arachidonic acid (ARA), stearidonic acid (SDA), eicosapentaenoic acid (EPA) and their precursors (linoleic acid [LIN], α‐linolenic acid [ALA], SDA, respectively) within *Daphnia* in each diet treatment.

PUFA in *Daphnia*	Δ^13^C_ARA_	Δ^13^C_SDA_	Δ^13^C_EPA_
*Planktothrix* diet			
LIN	−1.5 ‰ (n.s.)	–	–
ALA	–	+ 6.3 ‰ (*)	+ 0.7 ‰ (n.s.)
SDA	–	–	−5.6 ‰ (n.s.)
Chytrid‐infected *Planktothrix* diet			
LIN	−1.0 ‰ (n.s.)	–	–
ALA	–	+ 2.9 ‰ (n.s.)	−2.7 ‰ (n.s.)
SDA	–	–	−3.8 ‰ (n.s.)

*Note*: Positive numbers indicate *δ* ^13^C enrichment in polyunsaturated fatty acids relative to the precursor. The larger the number, the larger the isotopic fractionation. The level of significance is based on ANOVA, multiple comparisons of means, Tukey. n.s., not significant, *p < 0.05, **p < 0.01, ***p < 0.001, significant data are in bold.

## 4 DISCUSSION

The results supported our hypothesis that chytrids, as an intermediate‐level functional group, would enhance the fitness of the key herbivore zooplankton, *Daphnia*. Increased fitness in *Daphnia* could not result solely from the quantitative increase of ingested diet with chytrids. Rather, diet including chytrids unproportionally enhanced carbon yield in *Daphnia*, indicating the importance of the biochemical composition of ingested carbon. The increased availability of required PUFA due to the chytrid‐mediated carbon upgrading (Gerphagnon et al., 2019; Kagami et al., 2007; Rasconi et al., 2020; Taube et al., 2019) thus extends to *Daphnia*, underlying the enhanced fitness of the consumer.

### 4.1 Quantitative trophic transfer

Chytrids as an intermediate‐level functional group enhanced the dietary energy acquisition of *Daphnia* as evidenced by the recorded twofold increase in diet ingestion rate of the consumer. The chytrid infection itself, however, did not shorten the length of *Planktothrix* filaments (i.e., data on chytrid‐infected *Planktothrix* culture—diet), as has been argued and reported (Agha et al., 2016; Gerphagnon et al., 2013), which *mechanistic fragmentation* (Gerphagnon et al., 2013) would enhance cyanobacteria ingestion and so herbivory (Frenken et al., 2020). Rather, our chytrid‐infected *Planktothrix* culture contained both long and short filaments in the diet supplied, and the fragmentation of long filaments seemed to occur after the mechanical interference with *Daphnia* (i.e., grazing effect observed in the *residual* of diet). Enhanced grazing with chytrid infection could reflect either the increased edibility of shorter filaments or the improved physiological status of consumers (Frenken et al., 2020). The disproportional increase in carbon yield relative to ingested carbon by *Daphnia* suggests that chytrids support *Daphnia* fitness. It may then result in a positive feedback loop between: (1) increased somatic growth of the consumer in response to enhanced ingestion of the higher quality diet; and (2) the better performing *Daphnia* being able to graze more efficiently and so further ingest the diet of higher quality. Increased diet ingestion, therefore, also resulted from enhanced *Daphnia* fitness when feeding on the chytrid‐infected diet. The existence of a positive feedback loop is supported by:

1. the enhanced efficiency in diet ingestion with chytrids by the consumer over time (i.e. exponential vs. linear increase for chytrid‐infected and uninfected *Planktothrix*, respectively). Whether and how far the enhanced grazing efficiency could also result from a suppressed physiological status of the host with chytrid infection, or it was mainly related to the increased fitness of *Daphnia*, remains to be explored.
2. more efficient breaking‐up of *Planktothrix* by the *Daphnia* over time (i.e., significantly shorter filaments in the residual of the chytrid‐infected diet).
3. the increasing efficiency of *Daphnia* to control the chytrid infection with time (i.e., increasing efficiency to reduce chytrid prevalence). How proportionally this reduction resulted from the breaking‐up of the filaments, i.e. grazing of the chytrid‐infected end of the filaments, compared with the effect of increasing grazing rate on the zoospores, also remains to be explored.

The reproduction of *Daphnia* in the chytrid‐infected diet treatment was also related to *Daphnia* size, where the chytrids' positive dietary effect may manifest in a hierarchic way, i.e., first enhanced survival, then enhanced growth, and finally successful reproduction. Such hierarchy during somatic *Daphnia* development might also be visible in Agha et al. (2016), where diet‐related treatment effects in *Daphnia* appeared mainly on reproduction, while survival and body size of adults were less affected. This might be related to the overall higher dietary C supplied via shorter dietary *Planktothrix* filaments in their experiment, and so higher dietary carbon provision. Chytrids ensuring *Daphnia* reproduction under a *cyanobacterium bloom* (*Planktothrix*) have already been suggested experimentally. Agha et al. (2016) showed that *c*. 2,000–2,500 chytrid zoospores per ml enhanced *Daphnia* fitness and reproduction. Our chytrid zoospore density was considerably lower (*c*. 600 ind/ml) but could already enhance *Daphnia* fitness substantially. This may also have direct relevance to observational data where natural chytrid zoospore density can reach *c*. 3,000 ind/ml (see Rasconi et al., 2012; Sime‐Ngando, 2012). Sustained cladoceran growth has been evidenced during cyanobacteria blooms (Davis et al., 2012; Moustaka‐Gouni et al., 2006), suggesting the existence and importance of alternative energy pathways. As trophic transfer efficiency between phytoplankton and zooplankton decreases with cyanobacteria dominance (McCauley & Kalff, 1981; Selmeczy et al., 2019) and also the dominance of other large‐sized colonial and filamentous algae (Kagami et al., 2007, 2014), our results also support the *mycoloop hypothesis* of parasitic chytrids providing the energetic needs of zooplankton for survival, growth, and reproduction.

### 4.2 Qualitative trophic transfer

Dietary phosphorus could have potentially driven the treatment‐specific differences in life history traits of *Daphnia*. Enhanced *Daphnia* fitness, however, may not be explained by the mere elemental composition of the diet sources since *Daphnia* did not differ significantly in their respective C:P, N:P, and C:N ratios between diet treatments. Furthermore, the C:P ratio of *Daphnia* remain well‐below the potentially limiting C:P *c*. 155 (Khattak et al., 2018), further emphasising that *Daphnia* were not P limited in any of the diet treatments. The N:P ratio of chytrids is expected to follow the ratio of the host, which affects zoospore production (Frenken et al., 2017). We did not measure dietary N:P, but lower N:P in *Daphnia* feeding on the chytrid‐infected diet might indicate higher P availability relative to N, yet no significant differences in N availability between diet treatments. The C:N ratios reported for diet sources (*c*. 5) vary little and are similar to those reported by Frenken et al. (2017). In line with Barranco et al. (2020), chytrids might have upgraded diet quality in terms of N, resulting in significantly lower C:N ratios in chytrid zoospores compared with the host. However, lowering C:N did not extend further to *Daphnia*, suggesting that *Daphnia* adjusted its C:N ratio irrespective of dietary supply. Organic matter leakage linked to chytrid infection and related higher bacterial abundance could also lead to lower C:N ratio and so diet quality increase for zooplankton (Barranco et al., 2020). Our results confirmed a higher bacterial abundance linked to chytrid‐infection (Agha et al., 2016; Klawonn et al., 2021; Senga et al., 2018). Ingested bacterial carbon, however, remained only a minor fraction of total dietary carbon. The fact that the *Daphnia* fatty acid profile did change irrespective of the diet treatments is in line with the observation that ingested bacterial carbon does not provide dietary energy for growth and certainly not for reproduction (Taipale et al., 2012). Using a similar *Planktothrix*‐chytrid‐*Daphnia* experimental setup, Agha et al. (2016) showed that bacterial diet reduced somatic growth, fecundity and offspring size compared with the sole *Planktothrix* and chytrid‐infected *Planktothrix* diets. Accordingly, functional carbon that affected *Daphnia* fitness may have been originated from the cyanobacteria and chytrids, while the diet response observed did not depend on bacteria. Furthermore, the significant C:N decrease in *Daphnia* compared with the neonates may suggest that dietary carbon rather than nitrogen availability was constrained, especially for *Daphnia* feeding on the sole *Planktothrix* diet (i.e. C:N < 5). The eventual C limitation of *Daphnia* therefore emphasises the paramount importance of the molecular composition of diet, and thus, the quality of the C transferred to zooplankton.

No or slight differences in bulk *δ* ^13^C and *δ* ^15^N among the host, chytrid‐infected host, and *Daphnia* have been reported before, suggesting a large variation in isotopic enrichment across host‐parasitic systems (Barranco et al., 2020, and references therein). Despite the high variation in our data, the results may highlight the limitations of bulk stable isotopes to estimate trophic positions and basal energy sources. As reported on a diatom–chytrid–*Keratella* system (Barranco et al., 2020), here we also showed that the host together with the chytrids tended to be at the highest trophic position, while the zooplankton at a lower level. Grey (2006) suggested that *inversed δ* ^15^N chains might occur if the consumers were not in isotopic equilibrium with the diet, and the ^15^ N value was taken up faster by the primary producer than by the subsequent consumer. Thus, temporal variations in uptake and assimilation also have to be taken into account. Alternatively, an inversed *δ* ^15^N chain can also indicate starvation of the consumer and reflect changes in the internal use of N (Barranco et al., 2020). Due to the low dietary carbon provided via the hardly palatable *Planktothrix*, energy limitation of *Daphnia* was evident, especially on the sole *Planktothrix* diet. Non‐significant differences in *Daphnia* bulk *δ* ^13^C may also suggest that diet sources overlapped isotopically, or multiple isotopic fractionations might have occurred across the tropic levels, being further constrained by differences in the palatability of diet sources.

The n‐3 PUFA EPA is key for *Daphnia* growth and reproduction (Becker & Boersma, 2003; Sikora et al., 2016), but was absent in the diet. When dietary PUFA compositions do not meet the physiological requirements of consumers, bioconversion of dietary precursors for target PUFA is necessary. Chytrid‐synthesised SDA (Gerphagnon et al., 2019; Rasconi et al., 2020), was selectively retained by *Daphnia*, leading to a significant treatment‐specific difference in SDA contents. We provide isotopic evidence that SDA was bioconverted and made available from ALA of *Planktothrix* (Gerphagnon et al., 2019; Rasconi et al., 2020) for *Daphnia*. The fact that *Daphnia* efficiently retained dietary SDA when feeding on the chytrid‐infected diet might point at its potential physiological importance for *Daphnia*. Interestingly, we did not find evidence that chytrids also performed upgrading to the ω‐6 PUFA ARA, suggesting the preference of chytrids for n‐3 PUFA upgrading, for reasons that require further studies. The lack of conversion from SDA to EPA may be due to the relatively short test time and/or that these chytrids are unable to convert SDA to longer‐chain n‐3 PUFA. Alternatively, we suggest that EPA was bioconverted from ALA by the *Daphnia* itself without any intermediate conversion support.

### 4.3 Compound specific ^13^C sheds light on carbon assimilation

The carbon limitation of *Daphnia* feeding on the sole *Planktothrix* diet and the higher carbon yield in *Daphnia* feeding on the chytrid‐infected diet demonstrate the paramount importance of the composition in dietary PUFA carbon. The higher carbon content assimilated with the chytrid‐infected diet evidences chytrids' positive dietary effect, also mediated by PUFA. Increased dietary PUFA provision as SDA, on top of the slightly more carbon ingested, may have a beneficial effect on *Daphnia* fitness. The selective retention of dietary SDA also coincided with an increased SDA:C ratio in *Daphnia* feeding on the chytrid‐infected diet (data not shown). The increased PUFA:C ratio indicates higher PUFA than C retention in *Daphnia*, while the dietary effects of chytrids in supporting the bioconversion to EPA by *Daphnia* still remains to be elucidated.

The bioconversion of ALA to EPA by *Daphnia* was supported by CSI data that suggested that the chytrid parasite also synthesised LIN, an essential PUFA known to be synthesised mainly by algae (Taipale et al., 2013). The isotopically significantly heavier LIN (and also ALA) in chytrid zoospores, as well as in *Daphnia* relative to the chytrid‐infected *Planktothrix*, further suggests the metabolic use of these PUFA by which isotopically heavier LIN and ALA were retained by *Daphnia*. The isotopically lighter *δ* ^13^C_SDA_ values also suggest SDA synthesis in the chytrid zoospore, which was subsequently selectively retained by *Daphnia*. While we did not find treatment‐specific differences in ARA retention of *Daphnia*, the isotopically lighter ARA compared to LIN also suggests the synthesis of these essential PUFA, irrespective of the two diet treatments. Similarly, the isotopically lighter EPA compared to ALA also suggests the endogenous EPA synthesis by *Daphnia*.

## 5 CONCLUSIONS

Our experiment provides isotopic evidence of trophic upgrading by chytrid fungal parasites, resulting in improved dietary PUFA provision and subsequently higher fitness of the herbivorous consumer *Daphnia*. This study demonstrates that carbon ingestion by *Daphnia* was only slightly enhanced with chytrid‐infected diet, but the carbon transfer efficiency increased disproportionally, suggesting that chytrid‐mediated trophic PUFA upgrading is crucial for trophic carbon transfer. The selective retention of the chytrid‐synthesised SDA together with the enhanced carbon yield suggests that dietary SDA may support the bioconversion to the LC‐PUFA EPA in *Daphnia*. Compound‐specific stable carbon isotope analysis outperformed the power of bulk *δ* ^13^C data in identifying the trophic pathways of key PUFA. LIN and ALA were also synthetised by the chytrid parasite, and directly assimilated by *Daphnia*. We further demonstrated the endogenous bioconversion of LIN to ARA and ALA to EPA, respectively, by the *Daphnia*. The quantitative role of chytrids in supporting the innate conversion of short chain to LC‐PUFA in *Daphnia* remains to be further elucidated, e.g. via ^13^C‐labelling of key functional PUFA. We note that in addition to PUFA, chytrids also produce sterols de novo (Gerphagnon et al., 2019; Kagami et al., 2007), mediating dietary sterol provision and thus positive dietary effects of chytrids on consumers. We conclude that chytrid‐mediated improved dietary PUFA extends to *Daphnia*, which enhances its performance when exposed to poor nutritional quality and hardly palatable cyanobacteria.

## AUTHOR CONTRIBUTIONS

Conceptualisation: S.R., M.J.K., R.P. Developing methods: S.R., A.A., M.J.K., R.P. Conducting the research: A.A. Data analysis. A.A. Data interpretation: A.A., M.J.K., S.R., R.P., M.P. Preparation of figures and tables: A.A. Writing: A.A. wrote the original draft and then all authors edited and commented on the drafts.

## FUNDING INFORMATION

This work was supported by the Austrian Science Fund (FWF Project P 30419‐B29).

## Supporting information


Appendix S1

## Data Availability

The data that support the findings of this study are available from the corresponding author upon reasonable request.

## References

[fwb14010-bib-0001] Agha, R. , Saebelfeld, M. , Manthey, C. , Rohrlack, T. , & Wolinska, J. (2016). Chytrid parasitism facilitates trophic transfer between bloom‐forming cyanobacteria and zooplankton (*Daphnia*). Scientific Reports, 6(1), 35039. 27733762 10.1038/srep35039 PMC5062065

[fwb14010-bib-0002] American Public Health Association . (1998). Standard methods for the examination of water and wastewater (pp. 3–37). Public Health Association, American Water Works Association, and Water Environment Federation.

[fwb14010-bib-0003] Arndt, H. (1993). Rotifers as predators on components of the microbial web (bacteria, heterotrophic flagellates, ciliates)—A review. In J. J. Gilbert , E. Lubzens , & M. R. Miracle (Eds.), Rotifer symposium VI (pp. 231–246). Springer.

[fwb14010-bib-0004] Arts, M. T. , Brett, M. T. , & Kainz, M. (2009). Lipids in aquatic ecosystems. Springer Science & Business Media.

[fwb14010-bib-0005] Barranco, V. S. , Van der Meer, M. T. , Kagami, M. , Van den Wyngaert, S. , Van de Waal, D. B. , Van Donk, E. , & Gsell, A. S. (2020). Trophic position, elemental ratios and nitrogen transfer in a planktonic host–parasite–consumer food chain including a fungal parasite. Oecologia, 194(4), 541–554. 32803339 10.1007/s00442-020-04721-w PMC7683484

[fwb14010-bib-0006] Becker, C. , & Boersma, M. (2003). Resource quality effects on life histories of *Daphnia* . Limnology and Oceanography, 48(2), 700–706.

[fwb14010-bib-0007] Bern, L. (1994). Particle selection over a broad size range by crustacean zooplankton. Freshwater Biology, 32(1), 105–112.

[fwb14010-bib-0008] Bottrell, H. H. , Duncan, A. , Gliwicz, Z. M. , Grygierek, E. , Herzig, A. , Hillbricht‐Ilkowska, A. , Kurasawa, H. , Larsson, P. , & Weglenska, T. (1976). A review of some problems in zooplankton production studies. Norwegian Journal of Zoology, 24, 419–456.

[fwb14010-bib-0009] Brett, M. T. , Kainz, M. J. , Taipale, S. , & Hari, S. (2009). Phytoplankton, not allochthonous carbon, sustains herbivorous zooplankton production. Proceedings of the National Academy of Sciences of the United States of America, 106, 21197–21201. 19934044 10.1073/pnas.0904129106 PMC2795543

[fwb14010-bib-0010] Carpenter, S. R. , Lathrop, R. C. , & Muñoz‐del‐Rio, A. (1993). Comparison of dynamic models for edible phytoplankton. Canadian Journal of Fisheries and Aquatic Sciences, 50(8), 1757–1767.

[fwb14010-bib-0011] Chiapella, A. M. , Kainz, M. J. , & Strecker, A. L. (2021). Fatty acid stable isotopes add clarity, but also complexity, to tracing energy pathways in aquatic food webs. Ecosphere, 12(2), e03360. 34900386 10.1002/ecs2.3360 PMC8641385

[fwb14010-bib-0012] Cook, H. W. , & McMaster, C. R. (2004). Fatty acid desaturation and chain elongation in eukaryotes. In D. E. Vance & J. E. Vance (Eds.), Biochemistry of lipids, lipoproteins and membranes (pp. 181–204). Elsevier.

[fwb14010-bib-0013] Copeman, L. A. , Parrish, C. C. , Brown, J. A. , & Harel, M. (2002). Effects of docosahexaenoic, eicosapentaenoic, and arachidonic acids on the early growth, survival, lipid composition and pigmentation of yellowtail flounder (*Limanda ferruginea*): a live food enrichment experiment. Aquaculture, 210, 285–304.

[fwb14010-bib-0014] Davis, T. W. , Koch, F. , Marcoval, M. A. , Wilhelm, S. W. , & Gobler, C. J. (2012). Mesozooplankton and microzooplankton grazing during cyanobacterial blooms in the western basin of Lake Erie. Harmful Algae, 15, 26–35.

[fwb14010-bib-0015] Ducklow, H. W. , & Carlson, C. A. (1992). Oceanic bacterial production. In K. C. Marshall (Ed.), Advances in microbial ecology (pp. 113–181). Springer.

[fwb14010-bib-0016] Elser, J. J. , Hayakawa, K. , & Urabe, J. (2001). Nutrient limitation reduces food quality for zooplankton: *Daphnia* response to seston phosphorus enrichment. Ecology, 82(3), 898–903.

[fwb14010-bib-0017] Frenken, T. , Wierenga, J. , Gsell, A. S. , van Donk, E. , Rohrlack, T. , & Van de Waal, D. B. (2017). Changes in N:P supply ratios affect the ecological stoichiometry of a toxic cyanobacterium and its fungal parasite. Frontiers in Microbiology, 8, 1015. 28634476 10.3389/fmicb.2017.01015 PMC5459933

[fwb14010-bib-0018] Frenken, T. , Wierenga, J. , van Donk, E. , Declerck, S. A. , de Senerpont Domis, L. N. , Rohrlack, T. , & Van de Waal, D. B. (2018). Fungal parasites of a toxic inedible cyanobacterium provide food to zooplankton. Limnology and Oceanography, 63(6), 2384–2393.

[fwb14010-bib-0019] Frenken, T. , Wolinska, J. , Tao, Y. , Rohrlack, T. , & Agha, R. (2020). Infection of filamentous phytoplankton by fungal parasites enhances herbivory in pelagic food webs. Limnology and Oceanography, 65(11), 2618–2626.

[fwb14010-bib-0020] Ger, K. A. , Urrutia‐Cordero, P. , Frost, P. C. , Hansson, L. A. , Sarnelle, O. , Wilson, A. E. , & Lürling, M. (2016). The interaction between cyanobacteria and zooplankton in a more eutrophic world. Harmful Algae, 54, 128–144. 28073472 10.1016/j.hal.2015.12.005

[fwb14010-bib-0021] Gerphagnon, M. , Agha, R. , Martin‐Creuzburg, D. , Bec, A. , Perriere, F. , Rad‐Menéndez, C. , Gachon, C. M. M. , & Wolinska, J. (2019). Comparison of sterol and fatty acid profiles of chytrids and their hosts reveals trophic upgrading of nutritionally inadequate phytoplankton by fungal parasites. Environmental Microbiology, 21(3), 949–958. 30507060 10.1111/1462-2920.14489

[fwb14010-bib-0022] Gerphagnon, M. , Latour, D. , Colombet, J. , & Sime‐Ngando, T. (2013). Fungal parasitism: Life cycle, dynamics and impact on cyanobacterial blooms. PLoS One, 8(4), e60894. 23593345 10.1371/journal.pone.0060894 PMC3625230

[fwb14010-bib-0023] Grey, J. (2006). The use of stable isotope analyses in freshwater ecology: Current awareness. Polish Journal of Ecology, 54(4), 563–584.

[fwb14010-bib-0024] Guillard, R. R. , & Lorenzen, C. J. (1972). Yellow‐green algae with chlorophyllide. Journal of Phycology, 8(1), 10–14.

[fwb14010-bib-0025] Gulati, R. , & Demott, W. (1997). The role of food quality for zooplankton: Remarks on the state‐of‐the‐art, perspectives and priorities. Freshwater Biology, 38(3), 753–768.

[fwb14010-bib-0026] Havens, K. E. (2008). Cyanobacteria blooms: Effects on aquatic ecosystems. In H. K. Hudnell (Ed.), Cyanobacterial harmful algal blooms: State of the science and research needs (pp. 733–747). Springer. 10.1007/978-0-387-75865-7_33 18461790

[fwb14010-bib-0027] Heissenberger, M. , Watzke, J. , & Kainz, M. J. (2010). Effect of nutrition on fatty acid profiles of riverine, lacustrine, and aquaculture‐raised salmonids of pre‐alpine habitats. Hydrobiologia, 650(1), 243–254.

[fwb14010-bib-0029] Kagami, M. , Miki, T. , & Takimoto, G. (2014). Mycoloop: Chytrids in aquatic food webs. Frontiers in Microbiology, 5, 166. 24795703 10.3389/fmicb.2014.00166 PMC4001071

[fwb14010-bib-0030] Kagami, M. , von Elert, E. , Ibelings, B. W. , de Bruin, A. , & Van Donk, E. (2007). The parasitic chytrid, *Zygorhizidium*, facilitates the growth of the cladoceran zooplankter, *Daphnia*, in cultures of the inedible alga, *Asterionella* . Proceedings of the Royal Society B: Biological Sciences, 274(1617), 1561–1566. 10.1098/rspb.2007.0425 PMC2176168 17439852

[fwb14010-bib-0031] Khattak, H. K. , Prater, C. , Wagner, N. D. , & Frost, P. C. (2018). The threshold elemental ratio of carbon and phosphorus of *Daphnia magna* and its connection to animal growth. Scientific Reports, 8(1), 9673. 29946166 10.1038/s41598-018-27758-7 PMC6018670

[fwb14010-bib-0032] Klawonn, I. , Van den Wyngaert, S. , Parada, A. E. , Arandia‐Gorostidi, N. , Whitehouse, M. J. , Grossart, H. P. , & Dekas, A. E. (2021). Characterizing the “fungal shunt”: Parasitic fungi on diatoms affect carbon flow and bacterial communities in aquatic microbial food webs. Proceedings of the National Academy of Sciences of the United States of America, 118(23), e2102225118. 34074785 10.1073/pnas.2102225118 PMC8201943

[fwb14010-bib-0033] Klüttgen, B. , Dülmer, U. , Engels, M. , & Ratte, H. T. (1994). ADaM, an artificial freshwater for the culture of zooplankton. Water Research, 28(3), 743–746.

[fwb14010-bib-0034] Lampert, W. (1978). A field study on the dependence of the fecundity of *Daphnia* spec. On food concentration. Oecologia, 36(3), 363–369. 28309923 10.1007/BF00348062

[fwb14010-bib-0035] Leibold, M. A. (1989). Resource edibility and the effects of predators and productivity on the outcome of trophic interactions. The American Naturalist, 134(6), 922–949.

[fwb14010-bib-0036] Lund, J. W. G. , Kipling, C. , & Le Cren, E. D. (1958). The inverted microscope method of estimating algal numbers and the statistical basis of estimations by counting. Hydrobiologia, 11(2), 143–170.

[fwb14010-bib-0037] Martin‐Creuzburg, D. , Sperfeld, E. , & Wacker, A. (2009). Colimitation of a freshwater herbivore by sterols and polyunsaturated fatty acids. Proceedings of the Royal Society B: Biological Sciences, 276(1663), 1805–1814. 10.1098/rspb.2008.1540 PMC2674483 19324803

[fwb14010-bib-0038] McCauley, E. , & Kalff, J. (1981). Empirical relationships between phytoplankton and zooplankton biomass in lakes. Canadian Journal of Fisheries and Aquatic Sciences, 38(4), 458–463.

[fwb14010-bib-0039] Moustaka‐Gouni, M. , Vardaka, E. , Michaloudi, E. , Kormas, K. A. , Tryfon, E. , Mihalatou, H. , Gkelis, S. , & Lanaras, T. (2006). Plankton food web structure in a eutrophic polymictic lake with a history of toxic cyanobacterial blooms. Limnology and Oceanography, 51(1 Part 2), 715–727.

[fwb14010-bib-0040] Müller‐Navarra, D. C. (2008). Food web paradigms: The biochemical view on trophic interactions. International Review of Hydrobiology, 93(4–5), 489–505.

[fwb14010-bib-0041] Müller‐Navarra, D. C. , Brett, M. T. , Liston, A. M. , & Goldman, C. R. (2000). A highly unsaturated fatty acid predicts carbon transfer between primary producers and consumers. Nature, 403(6765), 74–77. 10638754 10.1038/47469

[fwb14010-bib-0042] Oksanen, F. , Blanchet, F. G. , Friendly, M. , Kindt, R. , Legendre, P. , McGlinn, D. , Minchin, P. R. , O'Hara, R. B. , Simpson, G. L. , Solymos, P. , Stevens, M. H. H. , Szoecs, E. , & Wagner, H. (2019). vegan: Community ecology package . R package version 2.5‐6.

[fwb14010-bib-0043] Pilecky, M. , Závorka, L. , Arts, M. T. , & Kainz, M. J. (2021). Omega‐3 PUFA profoundly affect neural, physiological, and behavioural competences – Implications for systemic changes in trophic interactions. Biological Reviews, 96, 2127–2145. 10.1111/brv.12747 34018324

[fwb14010-bib-0044] R Core Team . (2019). R: A language and environment for statistical computing. R Foundation for Statistical Computing.

[fwb14010-bib-0045] Rasconi, S. , Grami, B. , Niquil, N. , Jobard, M. , & Sime‐Ngando, T. (2014). Parasitic chytrids sustain zooplankton growth during inedible algal bloom. Frontiers in Microbiology, 5, 229. 24904543 10.3389/fmicb.2014.00229 PMC4033230

[fwb14010-bib-0046] Rasconi, S. , Niquil, N. , & Sime‐Ngando, T. (2012). Phytoplankton chytridiomycosis: Community structure and infectivity of fungal parasites in aquatic ecosystems. Environmental Microbiology, 14(8), 2151–2170. 22309120 10.1111/j.1462-2920.2011.02690.x

[fwb14010-bib-0047] Rasconi, S. , Ptacnik, R. , Danner, S. , Van den Wyngaert, S. , Rohrlack, T. , Pilecky, M. , & Kainz, M. J. (2020). Parasitic chytrids upgrade and convey primary produced carbon during inedible algae proliferation. Protist, 171(5), 125768. 33126022 10.1016/j.protis.2020.125768

[fwb14010-bib-1046] Ruess, L. , & Müller‐Navarra, D. C (2019). Essential biomolecules in food webs. Frontiers in Ecology and Evolution, 7, 269.

[fwb14010-bib-0048] Selmeczy, G. B. , Abonyi, A. , Krienitz, L. , Kasprzak, P. , Casper, P. , Telcs, A. , Somogyvári, Z. , & Padisák, J. (2019). Old sins have long shadows: Climate change weakens efficiency of trophic coupling of phyto‐and zooplankton in a deep oligo‐mesotrophic lowland lake (Stechlin, Germany)—A causality analysis. Hydrobiologia, 831(1), 101–117.

[fwb14010-bib-0049] Senga, Y. , Yabe, S. , Nakamura, T. , & Kagami, M. (2018). Influence of parasitic chytrids on the quantity and quality of algal dissolved organic matter (AOM). Water Research, 145, 346–353. 30170302 10.1016/j.watres.2018.08.037

[fwb14010-bib-0050] Sikora, A. B. , Petzoldt, T. , Dawidowicz, P. , & von Elert, E. (2016). Demands of eicosapentaenoic acid (EPA) in *Daphnia*: Are they dependent on body size? Oecologia, 182(2), 405–417. 27345442 10.1007/s00442-016-3675-5 PMC5021750

[fwb14010-bib-0051] Sime‐Ngando, T. (2012). Phytoplankton chytridiomycosis: Fungal parasites of phytoplankton and their imprints on the food web dynamics. Frontiers in Microbiology, 3, 361. 23091469 10.3389/fmicb.2012.00361 PMC3469839

[fwb14010-bib-0052] Sønstebø, J. H. , & Rohrlack, T. (2011). Possible implications of chytrid parasitism for population subdivision in freshwater cyanobacteria of the genus *Planktothrix* . Applied and Environmental Microbiology, 77(4), 1344–1351. 21169434 10.1128/AEM.02153-10 PMC3067206

[fwb14010-bib-0053] Taipale, S. , Strandberg, U. , Peltomaa, E. , Galloway, A. W. , Ojala, A. , & Brett, M. T. (2013). Fatty acid composition as biomarkers of freshwater microalgae: Analysis of 37 strains of microalgae in 22 genera and in seven classes. Aquatic Microbial Ecology, 71(2), 165–178.

[fwb14010-bib-0054] Taipale, S. J. , Brett, M. T. , Hahn, M. W. , Martin‐Creuzburg, D. , Yeung, S. , Hiltunen, M. , Strandberg, U. , & Kankaala, P. (2014). Differing *Daphnia* magna assimilation efficiencies for terrestrial, bacterial, and algal carbon and fatty acids. Ecology, 95(2), 563–576. 24669748 10.1890/13-0650.1

[fwb14010-bib-0055] Taipale, S. J. , Brett, M. T. , Pulkkinen, K. , & Kainz, M. J. (2012). The influence of bacteria‐dominated diets on *Daphnia magna* somatic growth, reproduction, and lipid composition. FEMS Microbiology Ecology, 82(1), 50–62. 22564190 10.1111/j.1574-6941.2012.01406.x

[fwb14010-bib-0056] Taube, R. , Fabian, J. , Van den Wyngaert, S. , Agha, R. , Baschien, C. , Gerphagnon, M. , Kagami, M. , Krüger, A. , & Premke, K. (2019). Potentials and limitations of quantification of fungi in freshwater environments based on PLFA profiles. Fungal Ecology, 41, 256–268.

[fwb14010-bib-0057] Twining, C. W. , Bernhardt, J. R. , Derry, A. M. , Hudson, C. M. , Ishikawa, A. , Kabeya, N. , Kainz, M. J. , Kitano, J. , Kowarik, C. , Ladd, S. N. , Leal, M. C. , Scharnweber, K. , Shipley, J. R. , & Matthews, B. (2021). The evolutionary ecology of fatty‐acid variation: Implications for consumer adaptation and diversification. Ecology Letters, 24, 1709–1731. 34114320 10.1111/ele.13771

[fwb14010-bib-0058] Twining, C. W. , Taipale, S. J. , Ruess, L. , Bec, A. , Martin‐Creuzburg, D. , & Kainz, M. J. (2020). Stable isotopes of fatty acids: Current and future perspectives for advancing trophic ecology. Philosophical Transactions of the Royal Society B, 375(1804), 20190641. 10.1098/rstb.2019.0641 PMC7333957 32536315

[fwb14010-bib-0059] Utermöhl, H. (1958). Zur vervollkommnung der quantitativen phytoplankton‐methodik: Mit 1 Tabelle und 15 abbildungen im Text und auf 1 Tafel. Internationale Vereinigung für theoretische und angewandte Limnologie: Mitteilungen, 9(1), 1–38.

[fwb14010-bib-0060] Wood, S. N. (2011). Fast stable restricted maximum likelihood and marginal likelihood estimation of semiparametric generalized linear models. Journal of the Royal Statistical Society (B), 73(1), 3–36.

[fwb14010-bib-0061] Wood, S. N. (2017). Generalized additive models: An introduction with R (2nd ed.). Chapman and Hall/CRC.

