# Supplementary material for: Chytrids enhance Daphnia fitness by selectively retained chytrid‐synthesised stearidonic acid and conversion of short‐chain to long‐chain polyunsaturated fatty acids
Source: Freshw Biol. 2022 Nov 12;68(1):77–90. doi: 10.1111/fwb.14010 (PMC10099718; doi:10.1111/fwb.14010)
Supplement: Supplementary file 1 — Appendix S1 [file FWB-68-77-s001.docx]

**Supplementary materials for:**

**Chytrids enhance *Daphnia* fitness by selectively retained chytrid-synthesised stearidonic acid and conversion of short-chain to long-chain polyunsaturated fatty acids**

Running head: Chytrids enhance PUFA provision for Daphnia

^1,3,#^ András Abonyi, [abonyi.andras@ecolres.hu](mailto:abonyi.andras@ecolres.hu)

^1,2^ Serena Rasconi, [serena.rasconi@inrae.fr](mailto:serena.rasconi@inrae.fr)

^1^ Robert Ptacnik, [robert.ptacnik@wcl.ac.at](mailto:robert.ptacnik@wcl.ac.at)

^1,4^ Matthias Pilecky, [matthias.pilecky@donau-uni.ac.at](mailto:matthias.pilecky@donau-uni.ac.at)

^1,4^ Martin J. Kainz, [martin.kainz@donau-uni.ac.at](mailto:martin.kainz@donau-uni.ac.at)

**
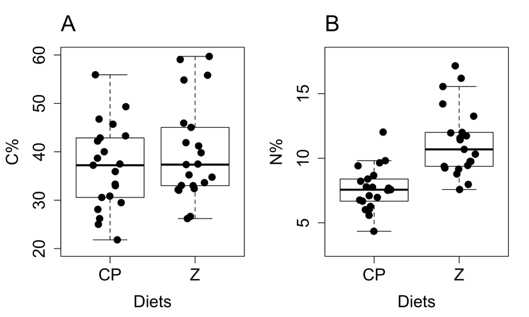
**

**Figure S1 (A)** Carbon content (%) of chytrid-infected *Planktothrix* (CP) and chytrid zoospores (Z); **(B)** Nitrogen content (%) of chytrid-infected *Planktothrix* (CP) and chytrid zoospores (Z). Preliminary analysis on the 8/3/2019 (n=21).

**Figure S2** Development of *Planktothrix* filament length over the course of the feeding experiment. Dark symbols show the filament length at the time of diet addition (‘diet’), light colours indicate the length of the residual (‘residual’) when food was replaced after 48 hours. **(A)** uninfected *Planktothrix* diet treatment, **(B)** chytrid-infected *Planktothrix* diet treatment

**Figure S3** **(A)** Prevalence (% of infected filaments) of chytrid infection in the diet supplied (chocolate) and in the residual after 48 hours (orange). Fitted lines are GAM (R^2^_adj_=0.131, p=n.s. and R^2^_adj_=0.547, p<0.001, for diet and residuals, respectively); **(B)** The difference in the prevalence of chytrids between the diet provided and its residual based on fitted linear trend line (LM, R^2^_adj_=0.715, p<0.001), where more negative the ∆ value, the lower the prevalence in the residual of the diet compared with the diet supplied.

**Figure S4** Mean ± SD for stable carbon (*δ*^13^C) and nitrogen (*δ*^15^N) isotope values of diet sources: cyanobacterium *Planktothrix* (‘CY’), chytrid-infected *Planktothrix* (‘CP’), and chytrid zoospores (‘Z’) and *Daphnia* consumers: *Daphnia* feeding on *Planktothrix* (‘D_CY’), and *Daphnia* feeding on chytrid-infected *Planktothrix* including chytrid zoospores (‘D_APZ’)

**Table S1** Elemental composition (µg mg^-1^ dry-weight) and molar ratios of diet sources and *Daphnia* consumer. Superscript letters denote the level of significance among diet sources, *Daphnia* neonates, and *Daphnia* in the different diet treatments based on ANOVA, Multiple comparison of means, Tukey (p<0.05).

| **Treatments** | **C** | **N** | **P** | **C:N** | **C:P** | **N:P** |
| --- | --- | --- | --- | --- | --- | --- |
| **Diet** |  |  |  |  |  |  |
| *Planktothrix* | 380±27 ^ab^ | 83±4 ^ab^ | *NA* | 5.4±0.2 ^ab^ | *NA* | *NA* |
| chytrid-infected *Planktothrix* | 348±46 ^a^ | 74±22 ^a^ | *NA* | 5.5±0.3 ^a^ | *NA* | *NA* |
| chytrid zoospores | 439±34 ^b^ | 100±6 ^b^ | *NA* | 5.1±0.1 ^b^ | *NA* | *NA* |
| ***Daphnia*** |  |  |  |  |  |  |
| neonates | 293±10 ^a^ | 51±2 ^a^ | 19±4 ^a^ | 7±0.1 ^a^ | 42±8 ^a^ | 6±1 ^a^ |
| Feeding *Planktothrix* | 240±18 ^a^ | 63±2 ^a^ | 12±4 ^a^ | 4±0.2 ^b^ | 55±14 ^a^ | 13±4 ^b^ |
| Feeding chytrid-infected *Planktothrix* | 280±41 ^a^ | 64±12 ^a^ | 17±1 ^a^ | 5±0.7 ^b^ | 43±5 ^a^ | 8±1 ^ab^ |
